# RNA sensor response in HeLa cells for transfected mRNAs prepared *in vitro* by SP6 and HiT7 RNA polymerases: A comparative study

**DOI:** 10.3389/fbioe.2022.1017934

**Published:** 2022-11-03

**Authors:** Siranjeevi Nagaraj, Anna Stankiewicz-Drogon, Edward Darzynkiewicz, Renata Grzela

**Affiliations:** ^1^ Interdisciplinary Laboratory of Molecular Biology and Biophysics, Centre of New Technologies, University of Warsaw Warsaw, Poland; ^2^ Division of Biophysics, Institute of Experimental Physics, Faculty of Physics, University of Warsaw Warsaw, Poland

**Keywords:** RNA polymerase, RNA sensor, MDA5, RIG-1, mRNA

## Abstract

*In vitro* transcribed (IVT) synthetic mRNAs are in high demand due to their attractive bench to clinic translational processes. Mainly, the procedure to make IVT mRNA using bacteriophage RNA polymerases (RNAP) is relatively uncomplicated and scalable to produce large quantities in a short time period. However, IVT mRNA preparations are accompanied by contaminants such as double-stranded RNA (dsRNA) as by-products that elicit undesired cellular immune responses upon transfections. Therefore, removing dsRNA contaminants is critical in IVT mRNA preparations for therapeutic applications. One such method to minimize dsRNA contaminants is to use genetically modified thermostable bacteriophage polymerase, HiT7 RNAP that performs IVT reaction at a higher temperature than typically used. However, the cellular RNA sensor response for IVT mRNA preparations by HiT7 RNAP is not characterized. Here, we compared the cellular RNA sensor response for mRNAs prepared by HiT7 RNAP (at 50°C) and SP6 RNAP (at 37°C) in HeLa cells. We show that IVT mRNA preparations by HiT7 RNAP reduced the dsRNA levels and dsRNA specific RNA sensor response (retinoic acid-inducible gene I, RIG-I and melanoma differentiation-associated 5, MDA5) compared to the IVT mRNA preparations by SP6 RNAP. Similarly, the incorporation of pseudouridine nucleotides instead of uridine nucleotides reduced dsRNA sensor response and increased the mRNA translation. Overall, the least dsRNA mediated RNA sensor response is observed when mRNA is synthesized by HiT7 RNAP and incorporated with pseudouridine nucleotides.

## Introduction

**FIGURE 1:**
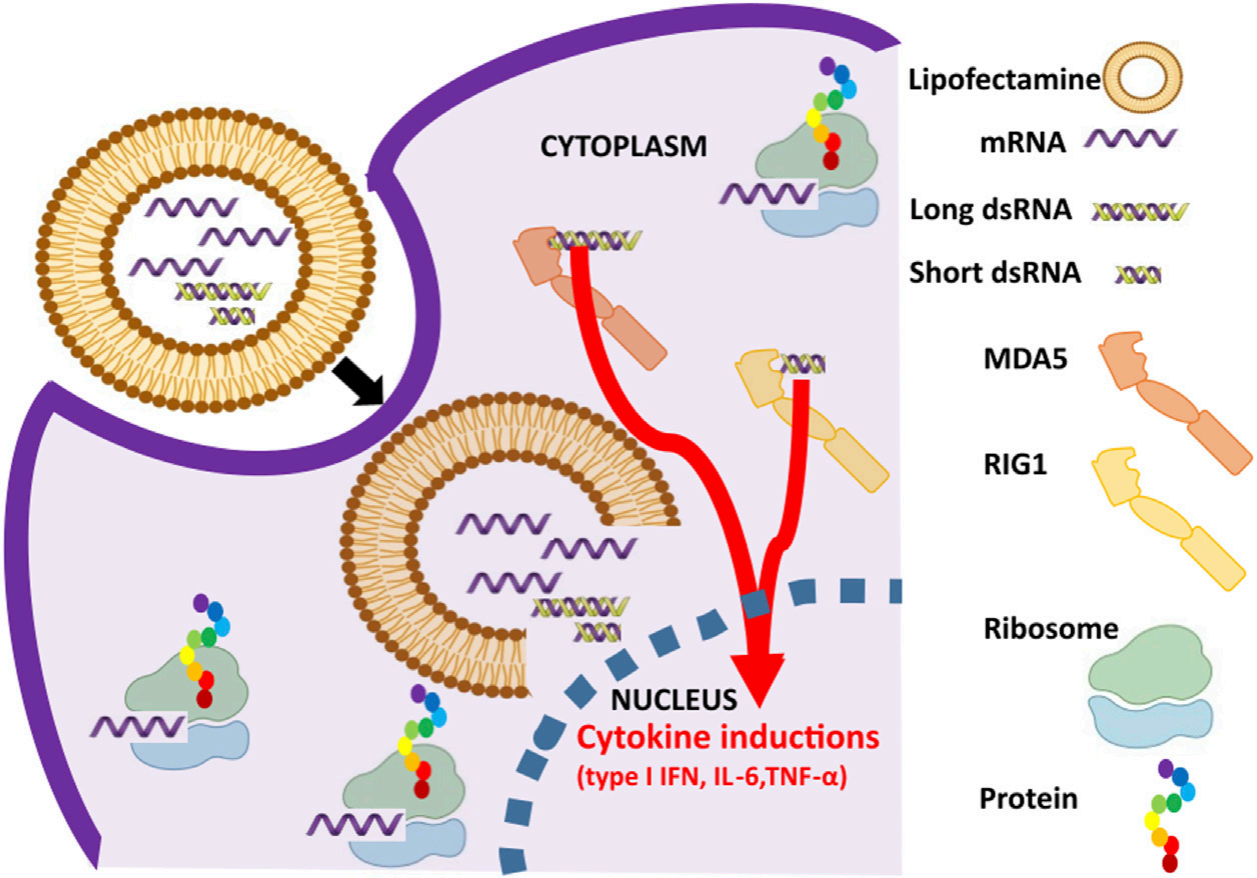
Activation of dsRNA sensor pathway in response to *in vitro* transcribed (IVT) mRNA preparations. mRNA and dsRNA impurities in the IVT mRNA preparations are lipocomplexed and transfected into the cells. While mRNA undergoes cap dependent translation in ribosomes, dsRNA impurities are bound by dsRNA specific RNA sensors such as RIG-I (sensing short dsRNA, shown in yellow) and MDA5 (sensing long dsRNA, shown in orange) to cause cytokine inductions such as type 1 interferon (IFN), interleukin-6 (IL-6), and tumor necrosis factor-α (TNF-α).

In recent times, the world has witnessed the proof of concept of mRNA based medicines in the form of SARS-CoV-2 vaccines (Szabó et al., 2022). Currently, there is growing interest in generating synthetic mRNAs for various therapeutic applications such as cancer immunotherapies, infectious disease vaccines, protein replacement therapies, regenerative medicine, and cellular reprogramming (Sahin et al., 2014; Grudzien-Nogalska et al., 2013; Damase et al., 2021; Chaudhary et al., 2021). Importantly, procedures to prepare IVT mRNAs are relatively simple and straightforward, making IVT mRNAs attractive drug molecules (Pardi et al., 2020). However, if IVT mRNAs are not prepared suitably, the introduction of mRNAs by lipid nanoparticles (LNP) mediated delivery onto cells (both *in vitro* and *in vivo*) causes adverse immune response by cytokine inductions (Mu et al., 2018; Mu and Hur, 2021; Patel et al., 2017) (Figure 1). Accumulating evidence suggest elicited undesired response is due to two factors: 1) unmodified nucleotide incorporation in the IVT prepared mRNAs and 2) contaminants, mainly dsRNA formed by either 3′-extended RNA or antisense RNA, that co-exist along with the prepared IVT mRNAs (Karikó et al., 2005; Karikó et al., 2008; Kormann et al., 2011; Nelson et al., 2020). The risk posed by unmodified nucleotides is overcome by incorporation of modified nucleotides containing pseudouridine, 1-methylpseudouridine, 6-methyladenosine, and 5-methylcytosine (Mu et al., 2018; Melamed et al., 2022; Moradian et al., 2022) and risk posed by dsRNA impurities is minimized by ultra-purification steps such as High Performance Liquid Chromatography (HPLC) and cellulose mediated mRNA purification (Karikó et al., 2011; Baiersdörfer et al., 2019). Relatively, HPLC procedure is laborious, not scalable, not cost effective and importantly, it reduces the yield of the mRNA (Baiersdörfer et al., 2019). On the other hand, cellulose mediated mRNA purification is reliable, scalable, cost effective that has been preferred for industry grade mRNA preparations (Baiersdörfer et al., 2019). In addition to these methods, reducing magnesium ions in IVT reaction (Mu et al., 2018), annealing a DNA oligonucleotide (called Capture DNA) complementary to the 3′ end of the RNA (Gholamalipour et al., 2019), and employing thermostable RNAPs in the IVT reaction (Wu et al., 2020) were suggested to get rid of dsRNA impurities.

In industry for large scale-up processes, ideally prepared IVT mRNAs should contain minimal dsRNA by-products contaminants to facilitate cost effective downstream post synthesis purification process to yield superior mRNA quality. Recently, thermostable T7 RNAPs (HiT7) that perform IVT reaction at high temperature (50°C) have been suggested to efficiently reduce the amount of dsRNA formed due to 3′-extended RNA (Wu et al., 2020). In the same study, comparisons were made for immunostimulatory effects caused by the transfection of IVT mRNAs prepared by conventional mesophilic T7 RNAP and genetically modified thermostable T7 RNAP, HiT7 RNAP. Wu et al. concluded that mRNA prepared by HiT7 RNAP reduced cellular immunogenicity. Nevertheless, comparative studies were not performed with mRNAs prepared with unrelated bacteriophage polymerase other than T7 RNAP such as SP6 RNAP. Moreover, expression levels of cellular RNA sensor responses for IVT mRNAs prepared by HiT7 RNAP were not studied. It is well known that upon transfection several RNA sensors respond to the IVT mRNA (Karikó and Weissman, 2007; Chan and Jin, 2022). Among these, some are specific to by-product dsRNA and others recognize changes in the mRNA itself. Specifically, dsRNA in the mRNA preparations are sensed by endosomal sensor: Toll-like receptor 3 (TLR3) (Alexopoulou et al., 2001) and by cytosolic sensors: oligoadenylate synthetase 1 (OAS1), retinoic acid-inducible gene I (RIG-I) - sensor of short cytosolic dsRNA and melanoma differentiation-associated 5 (MDA5) - sensor of long cytosolic dsRNA (Saito and Gale, 2008; Barral et al., 2009; Donovan et al., 2013; Brisse and Ly, 2019). Whereas, differences in the 5′ terminal cap in the mRNA are sensed by interferon induced proteins with tetratricopeptide repeats (IFITs) to discriminate between self and non-self RNAs (Choi et al., 2018; Miedziak et al., 2020). Progress in understanding the therapeutic potential of IVT mRNAs in anti-viral and anti-cancer studies are relatively advanced compared to studies exploring potential role of IVT mRNAs in protein replacement therapies and regenerative medicine. In this regard, underpinning cellular RNA sensing for IVT mRNAs is important not only in immune cells but also in other non-immune cells for wide therapeutic applications.

To fill this gap of understanding, in this study we compared the cellular RNA sensor responses of mRNAs prepared by SP6 and HiT7 RNAP in non-immune cell line, Hela. For this purpose, we synthesized mRNAs with unmodified (uridine nucleoside), modified (pseudouridine nucleoside) and/or HPLC purification. We show that a combination of HiT7 RNAP and nucleotide modification optimally reduced the cellular RNA sensor responses and provided efficient reporter production in cell.

## Methods

### Cell culture

HeLa and HEK293T cells were grown in Dulbecco’s Minimum Essential Medium (DMEM) (Biowest) supplemented with 10% foetal bovine serum (FBS) (Sigma-Aldrich), 2 mM L-glutamine and 100 mg/ml penicillin/streptomycin. Cells were cultured at 37°C in a humidified atmosphere of 5% CO_2_.

### *In vitro* transcription

To visualise the formation of dsRNA by-products during mRNA synthesis, a PCR product containing first 250 nt of the firefly luciferase coding sequence with the SP6 or T7 promoter sequence, purified with the NucleoSpin Gel and PCR clean-up (Macherey-Nagel), was used as dsDNA template for IVT reactions. A standard transcription reaction contained: transcription buffer, 25 ng/μl of dsDNA template, 1 mM ATP/CTP/GTP/UTP or ψUTP, 0.5 U/µL of ribolock ribonuclease inhibitor (Thermo) and 1 U/µl of SP6 RNAP, 1 U/µl of T7 RNAP (Thermo) or 2.5 U/µl of HiT7 RNAP (NEB). The reaction mixture was incubated 4 h at 37°C for SP6 and T7 RNAP and 4 h at 50°C for HiT7 RNAP. Following incubation, 0.025 U/µl of DNaseI (Thermo) was added and further incubated for 20 min at 37°C to remove template DNA. The transcripts were purified using the NucleoSpin RNA clean-up (Macherey-Nagel) according to the manufacturer’s instructions.

**FIGURE 2:**
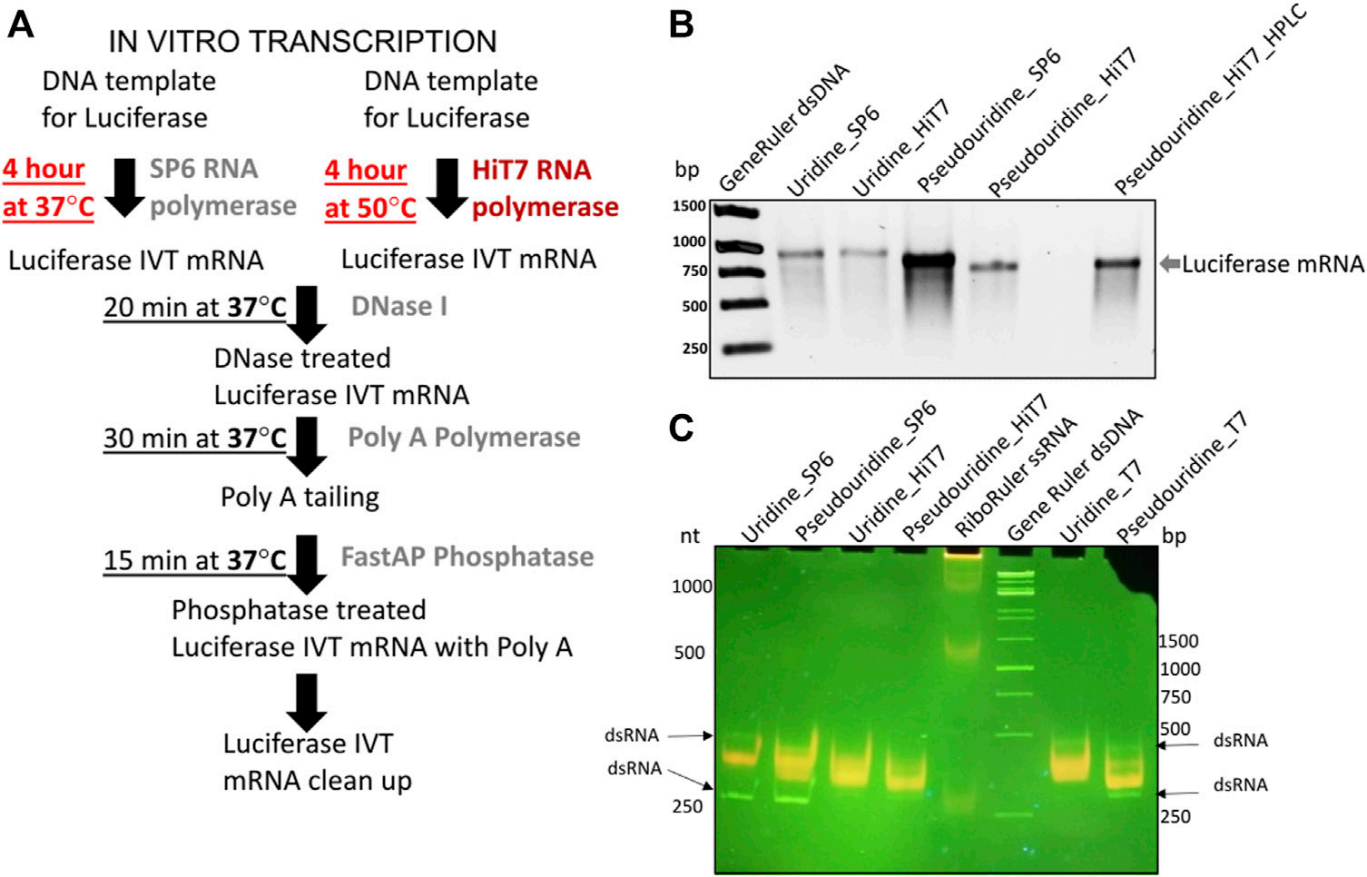
IVT firefly luciferase mRNA and short RNA prepared by different polymerase. **(A)** Shows the protocol for IVT reaction prepared by SP6 and HiT7 RNAPs **(B)** Shows different firefly luciferase mRNA preparations capped with 5′ m_2_^7,3’−O^GpppG (ARCA) incorporated with uridine/pseudouridine by SP6 and HiT7 RNAPs in 1% TAE agarose gel. Generuler dsDNA marker (in bp) was used as loading reference. **(C)** Shows different short RNA (250 nt) preparations incorporated with uridine/pseudouridine by SP6, T7 and HiT7 RNA polymerases in native 6% polyacrylamide 1X TAE gel. Acridine orange displaying different fluorescence upon binding to single strand RNAs (orange) and double strand RNAs (green). The Riboruler ssRNA marker (in nt) and Generuler dsDNA marker (in bp) was used as loading reference.

For transfection of HeLa and HEK293T cells, mRNAs were prepared in IVT reaction from a PCR template containing the firefly luciferase coding sequence with the SP6 or T7 promoter sequence, purified with the NucleoSpin Gel and PCR clean-up (Macherey-Nagel). RNA capping was carried out co-transcriptionally using cap analogue: m_2_^7,3’−O^GpppG (ARCA) (Stepinski et al., 2001) (molar ratio of cap:GTP was 10:1). A standard transcription reaction contained: transcription buffer, 25 ng/μl of dsDNA template, 1 mM ATP/CTP/UTP or ψUTP, 0.2 mM GTP, 2 mM dinucleotide ARCA cap analogue, 0.5 U/µl of ribolock ribonuclease inhibitor (Thermo Fisher Scientific) and 1 U/µl of SP6 RNAP or 2.5 U/µl of HiT7 RNAP. The reaction mixture was incubated 4 h at 37°C for SP6 RNAP and 4 h at 50°C for HiT7 RNAP. Following incubation, 0.025 U/µl of DNaseI (Thermo) was added and further incubated for 20 min at 37°C to remove template DNA. The whole reaction mixture was then subjected to 3′ end polyadenylation for 30 min at 37°C in a poly(A) buffer containing 1 mM ATP, 0.1 U/µl of poly(A) polymerase (NEB) and 0.4 U/µl of ribolock ribonuclease inhibitor. To remove free phosphate groups from the 5′ ends, transcripts were treated with alkaline phosphatase (FastAP, Thermo Fisher Scientific) for 15 min at 37°C in a reaction mixture containing FastAP buffer, 0.025 U/µl of FastAP and 0.33 U/µl of ribolock ribonuclease inhibitor. The transcripts were purified using the NucleoSpin RNA clean-up (Macherey-Nagel) according to the manufacturer’s instructions (Figure 2A). Quality of transcripts was checked on 1% 1X TAE agarose gels and concentration was measured spectrophotometrically (Figure 2B). To remove dsRNA by-products of IVT reaction by HPLC, mRNAs were purified on RNASep^TM^Prep–RNA Purification Column (ADS Biotec). A linear gradient of buffer B (25% acetornitrile in 0.1 M triethylammonium acetate pH 7.0) in buffer A (0.1 M triethylammonium acetate pH 7.0) from 35% to 55% for 20 min at flowrate 4 ml/min was applied. Fractions containing mRNA were concentrated on amicon ultra centrifugation filters (Merck), precipitated with sodium acetate and isopropanol mixture overnight at −20°C and precipitated RNA was dissolved in nuclease free water. The integrity of transcripts was checked on 1% 1X TAE agarose gel and concentration was determined spectrophotometrically.

### Visualisation of ss/dsRNA

To observe the formation of ss/dsRNAs during IVT, 0.5–1 μg of 250 nt-long mRNA was run on a native 6% polyacrylamide 1X TAE gel. The gel was stained post electrophoresis for 10 min either with SYBR™ Gold (Thermo Fisher Scientific) or with 25 μg/ml acridine orange hemi(zinc chloride) salt (AO, Sigma-Aldrich) in 1X TAE buffer, destained for 1 h to ON in water, and visualized on UV transilluminator.

### Transfection of firefly luciferase encoding mRNAs

In all these experiments, the passage number of HeLa cells was between 5 and 30, of HEK293T cells between 10 and 15. HeLa cells were seeded at 5 × 10^5^ cells/well in 24-well plates and grown to 60%–80% confluence 24 h prior to transfection. HEK293T cells were seeded at 2 × 10^4^ cells/well in 96-well plates coated with 200 μg/ml polyL-Lysine (Sigma-Aldrich) and grown to 60%–80% confluence 24 h prior to transfection. Cells were transfected with Lipofectamine™ 2000 transfection reagent according to the manufacturer’s protocol. Briefly, HeLa cells in each well were transfected using a mixture of 2 μl Lipofectamine™ 2000 transfection reagent and 250 ng mRNA encoding firefly luciferase in 500 μl of Opti-MEM. HEK293T cells were transfected with 100 ng RNA and 0.6 μl Lipofectamine™ 2000 transfection reagent in 10 μl of Opti-MEM per well containing 90 μl of DMEM. Cells were collected at 8 h and 24 h post transfection.

### Luciferase reporter assay

HEK293T cells transfected with 250 nt-long mRNAs were lysed by addition of 20 µl per well of Cell Culture Lysis Reagent (Promega). To estimate the luciferase activity, the lysates were mixed with the luciferase substrate (Promega, United States) in ratio 1:2 and the luminescence was measured on Synergy H1MFDG Microplate Reader (BioTek, United States). To avoid errors due to varying number of cells in each well, the luminescence values were normalized to the total protein concentration measured with Roti-Quant Protein quantitation assay according to Bradford (Roth, USA).

### RNA isolation and RT-qPCR

Total RNA was isolated from cells using the Total RNA Mini isolation kit (A&A Biotechnology) according to the manufacturer’s instructions. RNA was quantified spectrophotometrically and its quality was analyzed using A_260_/A_280_ ratio (DeNovix). Up to 150 ng of RNA was used to obtain cDNA using the High-Capacity cDNA Reverse Transcription Kit (Thermo Fisher Scientific). Quantitative PCR was performed on LightCycler 480 II System (Roche). Briefly, 2 μl of cDNA (obtained from 150 ng of RNA), 4 μl of each mRNA specific primers (5 pmol of forward and reverse) (Supplementary Table S1), 10 μl of Maxima SYBR Green qPCR Master Mix (2X) (Thermo Fisher Scientific) were mixed in a 20 μl reaction and run with a thermal profile of an initial 10 min melting step at 95°C, followed by 45 cycles at 95°C for 10 s, 60°C for 10 s and 72°C for 10 s. The relative fold change of mRNAs was normalized to β-actin mRNA by 2^−ΔΔCt^ method (Livak and Schmittgen, 2001).

### Statistical analysis

All statistical analyses were performed with R software v3.6.2 (https://cran.r-project.org). One-way ANOVA with post–hoc Tukey HSD test was used. Statistical significance in *p* value was denoted in asterixes (*p ≤ 0.05, **p ≤ 0.01,***p ≤ 0.001) and the data are shown as mean ± standard error for three to six independent replicates. In all experiments, the term independent replicates specify the same IVT prepared RNAs that are then used in independent cultures.

## Results

### mRNA preparations by SP6 and HiT7 RNAPs

In order to gain insight into the cellular RNA sensor responses to transfections of firefly luciferase IVT mRNAs, we prepared different mRNAs capped at the 5′ end with m_2_^7,3’−O^GpppG (anti-reverse cap analogue, ARCA) incorporating unmodified uridine/modified pseudouridine nucleotides and obtained by different RNAP (SP6 and HiT7) as shown in Figure 2B. To ease readership, we used short names for mRNAs based on their preparations: mRNA_Uridine_SP6 - uridine containing firefly luciferase IVT mRNA prepared by SP6 RNAP; mRNA_Uridine_HiT7 - uridine containing firefly luciferase IVT mRNA prepared by HiT7 RNAP; mRNA_Pseudouridine_SP6 - pseudouridine containing firefly luciferase IVT mRNA prepared by SP6 RNAP; mRNA_Pseudouridine_HiT7 - pseudouridine containing firefly luciferase IVT mRNA prepared by HiT7 RNAP; mRNA_Pseudouridine_HiT7_HPLC–pseudouridine containing firefly luciferase IVT mRNA prepared by HiT7 RNAP with post synthesis HPLC purification. We observed firefly luciferase mRNA yield was slightly higher in the IVT reaction performed by SP6 RNA polymerase compared to HiT7 RNAP (Figure 2B). Further, we conducted acridine orange dye binding assay using short RNAs (250 nt corresponding to N terminal part of luciferase) prepared by different RNAPs to assess the extent of dsRNA in the IVT reactions performed. Notably, short RNA prepared by HiT7 RNAP showed lower dsRNA levels compared to the short RNA prepared by SP6 and T7 RNAPs (Figure 2C).

### RNA sensor response for mRNA preparations

**FIGURE 3:**
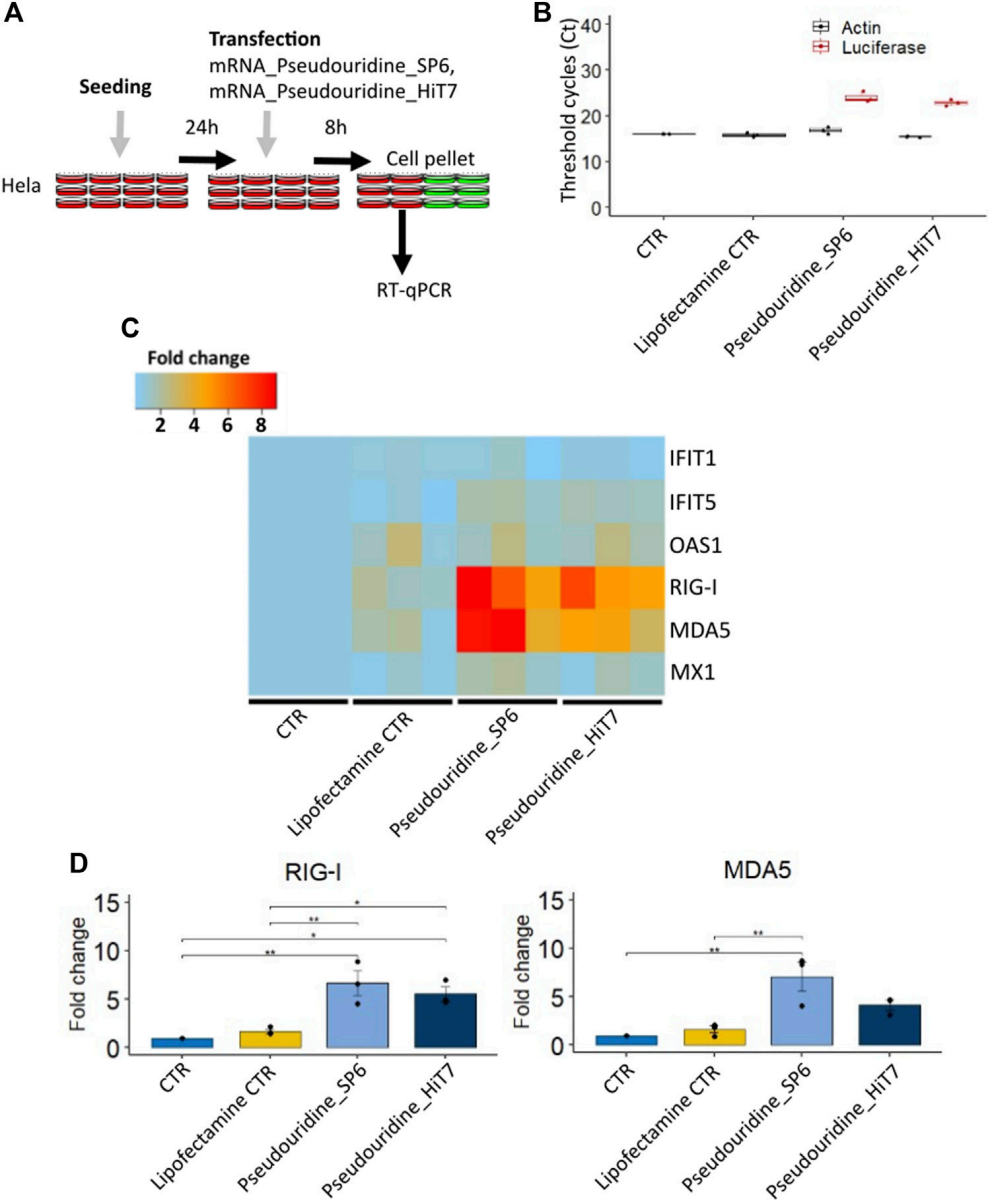
Cellular RNA sensor response of different firefly luciferase IVT mRNA prepared with pseudouridine incorporation by SP6 RNA polymerase and HiT7 RNA polymerase. **(A)** Shows the scheme of the experimental design **(B)** Confirmation of the transfection by detecting firefly luciferase mRNA by RT-qPCR. **(C)** Heat map showing *IFIT1, IFIT5, OAS1, MX1, RIG-I, MDA5* fold change levels determined by RT-qPCR at 8 h post transfection. **(D)** Bar chart showing *RIG-I* and *MDA5* fold change levels determined by RT-qPCR at 8 h post transfection. β-Actin (ACTB) was used as a reference in C and D.

To investigate if mRNA preparations by HiT7 RNAP would limit the cellular RNA sensor response triggered by contaminants, we transfected HeLa cells with different preparations containing mRNAs incorporated with modified pseudouridine nucleotides (mRNA_Pseudouridine_SP6 and mRNA_Pseudouridine_HiT7), collected cells at 8 h post transfection and measured the induction levels of RNA sensors (Figures 3A–D). Firstly, we confirmed the transfection of firefly luciferase IVT mRNA by RT-qPCR (Figure 3B). Secondly, we found no appreciable changes for IFITs (*IFIT1* and *IFIT5*) between mRNA_Pseudouridine_SP6 and mRNA_Pseudouridine_HiT7 transfections (Figure 3C). This is perhaps expected, since IFITs sensors bind to cap structure (preferentially to m^7^GpppN, cap 0), and therefore do not recognize dsRNA contaminants in the mRNA preparations. In addition, we did not notice induction of *OAS1* cytosolic dsRNA sensors 8 h post transfection with either of the transcripts (Figure 3C). However, we observed that cells transfected with mRNA_Pseudouridine_HiT7 showed slightly lower induction of *RIG-I*, sensor of short cytosolic dsRNA, and lower induction of *MDA5*, sensor of long cytosolic dsRNA, when compared to the cells transfected with mRNA_Pseudouridine_SP6 (Figures 3C,D). This observation reflects minimal dsRNA impurities co-produced when RNA is prepared by HiT7 RNAP and is consistent with the known function of cytosolic dsRNA sensors to bind and respond to dsRNA by increasing their fold change (Broquet et al., 2011; Bartok and Hartmann, 2020) (Figures 1, 2C). Of note, MX Dynamin Like GTPase 1 (*MX1*) sensor showed no striking changes (Figure 3C).

### dsRNA sensor response and luciferase translation for mRNA preparations: Uridine vs. pseudouridine modification

**FIGURE 4:**
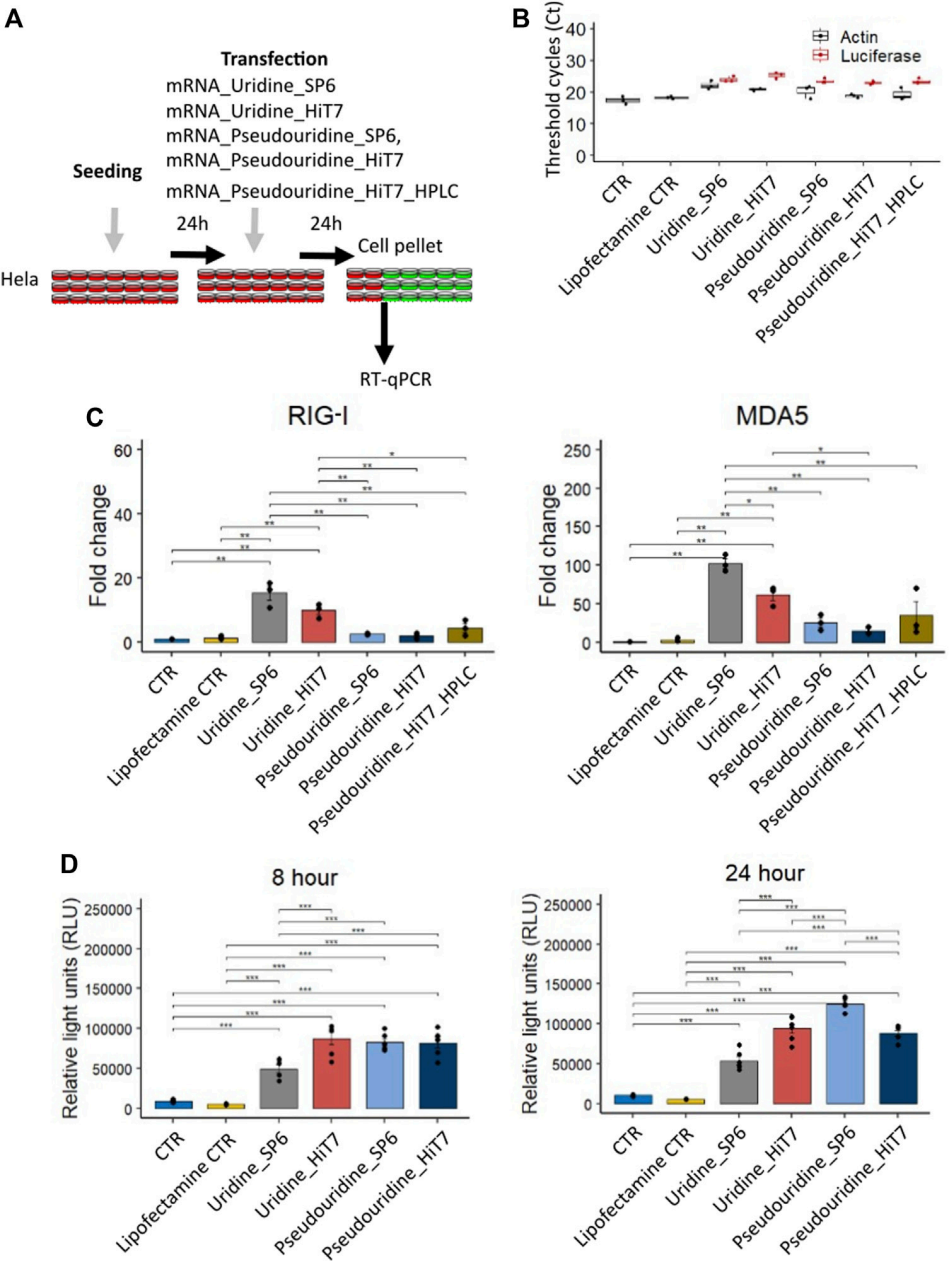
Cellular dsRNA sensor response and luciferase activity of different firefly luciferase IVT mRNA prepared with uridine/pseudouridine incorporation by SP6 RNA polymerase and HiT7 RNA polymerase. **(A)** Shows the scheme of the experimental design, **(B)** Confirmation of the transfection by detecting firefly luciferase mRNA by RT-qPCR. **(C)** Bar chart showing *RIG-I* and *MDA5* fold change levels determined by RT-qPCR at 24 h post transfection. β-Actin (*ACTB*) was used as a reference. **(D)** Bar chart showing luciferase activity in relative light units (RLU) for different firefly luciferase IVT mRNA preparations at 8 h and 24 h post transfection.

At 8 h post transfection we noticed that the cellular RNA sensor response was low and moreover, we lacked transfections with uridine containing mRNAs for comparison with mRNAs containing modified pseudouridine nucleotides. We, therefore, transfected HeLa cells with different RNA preparations (mRNA_Uridine_SP6, mRNA_Uridine_HiT7, mRNA_Pseudouridine_SP6, mRNA_Pseudouridine_HiT7, and mRNA_Pseudouridine_HiT7_HPLC), collected the cells at 24 h post transfection and measured the induction levels of RNA sensors specific for dsRNA (Figures 4A–C). In these transfected cells, we confirmed firefly luciferase IVT mRNA by RT-qPCR (Figure 4B). We anticipated that mRNAs prepared by HiT7 RNAP would reduce the amount of dsRNA contaminants, therefore would reduce the induction levels of cytosolic dsRNA sensors *RIG-I* and *MDA5* (Figures 3C,D) (Brisse and Ly, 2019). When comparing transfections with uridine containing mRNAs, as expected the cells transfected with mRNA_Uridine_HiT7 showed lower induction of *RIG-I* (not statistically) and of *MDA5* (statistically) when compared to the cells transfected with mRNA_Uridine_SP6 (Figure 4C). Similarly, when comparing transfections with pseudouridine containing mRNAs prepared by two different RNAPs, we noticed that the cells transfected with mRNA_Pseudouridine_HiT7 showed almost no change in the induction level of *RIG-I* and slightly lowered induction of *MDA5* (not statistically) when compared to the cells transfected with mRNA_Pseudouridine_SP6 (Figure 4C). Overall, when we used modified nucleotide pseudouridine instead of uridine for mRNA preparations, we noticed that the induction levels of *RIG-I* and *MDA5* were significantly lowered upon transfections. This phenomenon is observed for mRNAs prepared by both RNAPs: SP6 and HiT7 (Figure 4C). Next, we wanted to examine the influence of HPLC purification on dsRNA sensor response. We did not find HPLC purification further reduced the level of dsRNA sensor induction, on the contrary, it was slightly higher than observed for mRNA_Pseudouridine_SP6 and for mRNA_Pseudouridine_HiT7. Finally, we used luciferase reporter assay to evaluate the translation of different mRNA preparations by SP6 RNAP and HiT7 RNAP. At both time points (8 h and 24 h) reporter assay revealed, SP6 translation was significantly lowered compared to the mRNA_Uridine_HiT7, mRNA_Pseudouridine_SP6, mRNA_Pseudouridine_HiT7 (Figure 4D). This observation is consistent with the higher expression of *RIG-I* and *MDA5* in mRNA_Uridine_SP6 preparation compared to the mRNA_Uridine_HiT7, mRNA_Pseudouridine_SP6, mRNA_Pseudouridine_HiT7 (Figure 4C). Surprisingly, we noticed no significant difference in the translation levels of luciferase in cells transfected with mRNA_Uridine_HiT7 and mRNA_Pseudouridine_HiT7, despite significant changes observed in induction of cytosolic dsRNA sensor levels (Figures 4C,D). Moreover, not at 8 h but at 24 h, luciferase translation of mRNA_Pseudouridine_SP6 was slightly higher than mRNA_Pseudouridine_HiT7 (Figure 4D).

## Discussion

At present, RNA technology is experiencing rapid progress. A variety of therapies based on different types of RNA, such as mRNAs, small interfering RNAs, ribozymes and aptamers are being developed. All the above require efficient and inexpensive methods for producing RNA. While small molecules can be prepared by organic chemistry methods, large mRNAs require a different approach. For many years, the most popular method for mRNA preparation has been *in vitro* transcription (IVT) with the use of RNAPs, primarily T7 and SP6. Both of them show very high structural and functional similarity. However, some features distinguish them. Each polymerase is highly specific for its promoter, and the substrates required for mRNA synthesis are also different. While SP6 RNAP requires the complete pairing of DNA substrate, T7 RNAP only needs the pairing of a fragment of 18 bases (Stump and Hall, 1993). Most importantly for mRNA production, more product can be obtained, and the transcription process itself can be easily scaled up to milligram quantities when SP6 RNAP is used.

A new variant of T7 RNAP has recently appeared, namely HiT7 RNAP. It is specific to the T7 phage promoter, but was designed to carry out reactions at higher temperatures. It shows activity in the temperature range of 37–56°C with the optimum at 50–52°C. Higher reaction temperature affects the amount of by-products formed. Two main by-products generated during *in vitro* transcription are: 1) 3′-extended RNAs that anneal to complementary sequences in either *cis* or *trans* to form duplexes (Triana-Alonso et al., 1995; Gholamalipour et al., 2018) and 2) antisense RNAs that hybridize to the RNA molecule to form dsRNA (Mu et al., 2018). These by-products are unfavorable since dsRNA triggers an immune system response. Various methods for removing by-products have been developed. HPLC chromatography is one of the best approaches applied for IVT purification (Karikó et al., 2011). However, this is a laborious technique that does not guarantee the removal of all by-products. Another option is to use RNase III, specific for dsRNA (Foster et al., 2019). Although this solution is easy to implement, it carries disadvantages, because the nuclease is unable to distinguish dsRNA from single-stranded RNA with secondary structures (Baiersdörfer et al., 2019). This will particularly affect the production of long RNA, which contains more such structures and will be digested more frequently. Similarly purification on cellulose resin will remove both dsRNA and ssRNA containing secondary structures, leading to lower recovery rates (Baiersdörfer et al., 2019). Additional RNA purification steps after the transcription reaction represent a bottleneck in the preparation of the mRNA molecule. Therefore, the new variant of T7 RNAP, capable of generating reduced amount of dsRNA during IVT reaction is an extremely appealing alternative.

The mechanisms for the formation of by-products during *in vitro* mRNA synthesis have been proposed, and HiT7 RNAP has been shown to significantly reduce the amount of dsRNA when used at elevated temperatures (Wu et al., 2020). Nevertheless, data on the cellular RNA sensor response to HiT7 RNAP products are limited. The evidence available refers only to the secretion of IFN-α after the transfection of various T7 RNAP products into the cell. Therefore, we decided to further characterize the response of RNA sensors after the introduction of mRNA molecules prepared by HiT7 RNAP. Additionally, we included mRNA molecules prepared by SP6 RNAP for comparison. This enzyme allows for higher product yields than T7 RNAP and provides easy scalability of the reaction, making it of interest to pharmaceutical companies. Taken together, our data suggest that the mRNAs prepared by HiT7 RNAP show lower induction of cellular RNA sensors, previously shown to bind dsRNA, compared to mRNAs prepared by SP6 RNAP. Specifically, mRNA preparations by HiT7 RNAP significantly reduce the induction level of *MDA5*, sensor of long cytosolic dsRNA, when compared to mRNA preparations by SP6 RNAP.

We confirmed the presence of dsRNA by-products in the preparation of the transcripts using acridine orange dye, which emits orange fluorescence when bound to phosphate groups in ssRNA and green fluorescence when intercalated into dsRNA (Mu et al., 2018). As expected, short RNA prepared with the use of HiT7 RNAP had significantly less by-products compared to other samples.

Further, in our study we did not observe the beneficial effect of HPLC purification as post synthesis purification step when modified pseudouridine nucleotide was introduced (Figures 4C,D). In line with our observations, a recent study reported that modified nucleotides are sufficient to reduce RNA sensor response without additional HPLC purification step (Vaidyanathan et al., 2018). Of note, the presence of pseudouridine prevents the activation of RNA-dependent protein kinase (PKR), which leads to phosphorylation of eIF-2α and translational inhibition. This is due to weaker binding to PKR of pseudouridine-containing RNA compared to uridine-containing RNA (Anderson et al., 2010). Furthermore, corroborating our finding Wu et al. showed that there was no significant difference in the secreted IFN-α levels in the supernatant of dendritic cells upon transfection with *Cypridina* luciferase mRNA_Pseudouridine_HiT7 and *Cypridina* luciferase mRNA_Pseudouridine_HiT7_HPLC (Wu et al., 2020). To conclude, a combination of pseudouridine incorporation and transcription with HiT7 RNAP for mRNA preparations is needed to achieve the lowest induction of dsRNA-mediated cellular sensor response. We believe the approach demonstrated by our study is transferable to any RNA preparations irrespective of their sequence difference. Nevertheless, pseudouridine replacement suggested to increase the frequency of substitution errors during RNA synthesis by T7 RNAP (Potapov et al., 2018). Further understanding in these mechanistic insights are needed for better design of druggable mRNA molecules.

## Data Availability

The original contributions presented in the study are included in the article/Supplementary Material, further inquiries can be directed to the corresponding author.
