# Supplementary material for: RNA sensor response in HeLa cells for transfected mRNAs prepared in vitro by SP6 and HiT7 RNA polymerases: A comparative study
Source: Front Bioeng Biotechnol. 2022 Nov 3;10:1017934. doi: 10.3389/fbioe.2022.1017934 (PMC9669293; doi:10.3389/fbioe.2022.1017934)
Supplement: Supplementary file 1 [file Table1.pdf]

Target	Primer	Sequence (5’ to 3’)
IFIT1	Forward	CTGTGGTAGGCTCTGCTTCC
IFIT1	Reverse	AGCAGTCCACTCACCTCAGC
IFIT5	Forward	ACATGTAGGCTGAAGGAGGC
IFIT5	Reverse	CTTGTCAGTTTGGTGCGAAG
OAS1	Forward	CTCAAGAGCCTCATCCG
OAS1	Reverse	GCAGAGTTGCTGGTAGTTTA
MX1	Forward	ACAAGCGGAAGTTCCTGAAG
MX1	Reverse	GCACTCAAGTCGTCAGTCCA
RIG-I	Forward	GATCCCAGCAATGAGAATCC
RIG-I	Reverse	GCCACGTCCAGTCAATATGC
MDA5	Forward	GGTTGGACTCGGGAATTCGT
MDA5	Reverse	CTCAAACGATGGAGAGGGCA
ACTB	Forward	GCCGAGGACTTTGATTGC
ACTB	Reverse	CTGTGTGGACTTGGGAGAG
Luc 250 nt	Reverse	CCATATCGTTTCATAGCTTCTG

Supplementary table 1. Primers used in this study

RNA	dsDNA template sequence<br>(used primers are underlined, promoters are in bold)
short RNA_SP6	**ATTTAGGTGACACTATA**GAACCAGATCACTGTGTTTACTTGCAATCCCCCAAAACAGAC<br>AGAATGGAAGACGCCAAAAACATAAAGAAAGGCCCGGCGCCATTCTATCCGCTGGAAGATGG<br>AACCGCTGGAGAGCAACTGCATAAGGCTATGAAGAGATACGCCCTGGTTCCTGGAACAATTG<br>CTTTTACAGATGCACATATCGAGGTGGACATCACTTACGCTGAGTACTTCGAAATGTCCGTTCG<br>GTTGGCAGAAGCTATGAAACGATATGG
short RNA_T7	CAGACATATGCA**TAATACGACTCACTATA**GGGAACCAGATCACTGTGTTTACTTGC<br>AATCCCCCAAAACAGACAGAATGGAAGACGCCAAAAACATAAAGAAAGGCCCGGCGCCATT<br>CTATCCGCTGGAAGATGGAACCGCTGGAGAGCAACTGCATAAGGCTATGAAGAGATACGCCC<br>TGGTTCCTGGAACAATTGCTTTTACAGATGCACATATCGAGGTGGACATCACTTACGCTGAGTA<br>CTTCGAAATGTCCGTTCGGTTGGCAGAAGCTATGAAACGATATGG
short RNA_HiT7	CAGACATATGCA**TAATACGACTCACTATA**GGGAACCAGATCACTGTGTTTACTTGC<br>AATCCCCCAAAACAGACAGAATGGAAGACGCCAAAAACATAAAGAAAGGCCCGGCGCCATT<br>CTATCCGCTGGAAGATGGAACCGCTGGAGAGCAACTGCATAAGGCTATGAAGAGATACGCCC<br>TGGTTCCTGGAACAATTGCTTTTACAGATGCACATATCGAGGTGGACATCACTTACGCTGAGTA<br>CTTCGAAATGTCCGTTCGGTTGGCAGAAGCTATGAAACGATATGG
firefly luciferase IVT mRNA_SP6	**ATTTAGGTGACACTATA**GAACCAGATCACTGTGTTTACTTGCAATCCCCCAAAACAGAC<br>AGAATGGAAGACGCCAAAAACATAAAGAAAGGCCCGGCGCCATTCTATCCGCTGGAAGATGG<br>AACCGCTGGAGAGCAACTGCATAAGGCTATGAAGAGATACGCCCTGGTTCCTGGAACAATTG<br>CTTTTACAGATGCACATATCGAGGTGGACATCACTTACGCTGAGTACTTCGAAATGTCCGTTCG<br>GTTGGCAGAAGCTATGAAACGATATGGGCTGAATACAAATCACAGAATCGTCGTATGCAGTG<br>AAAACTCTCTTCAATTCTTTATGCCGGTGTTGGGCGCGTTATTTATCGGAGTTGCAGTTGCGCC<br>CGCGAACGACATTTATAATGAACGTGAATTGCTCAACAGTATGGGCATTTCGCAGCCTACCGT<br>GGTGTTCGTTTCCAAAAAGGGGTTGCAAAAAATTTTGAACGTGCAAAAAAAGCTCCCAATCAT<br>CCAAAAAATTATTATCATGGATTCTAAAACGGATTACCAGGGATTTCAGTCGATGTACACGTTC<br>GTCACATCTCATCTACCTCCCGGTTTTAATGAATACGATTTTGTGCCAGAGTCCTTCGATAGGG<br>ACAAGACAATTGCACTGATCATGAACTCCTCTGGATCTACTGGTCTGCCTAAAGGTGTCGCTCT<br>GCCTCATAGAACTGCCTGCGTGAGATTCTCGCATGCCAGAGATCCTATTTTTGGCAATCAAATC

	ATTCCGGATACTGCGATTTTAAGTGTTGTTCCATTCCATCACGGTTTTGGAATGTTTACTACACT<br>CGGATATTTGATATGTGGATTTCGAGTCGTCTTAATGTATAGATTTGAAGAAGAGCTGTTTCTG<br>AGGAGCCTTCAGGATTACAAGATTCAAAGTGCGCTGCTGGTGCCAACCCTATTCTCCTTCTTCG<br>CCAAAAGCACTCTGATTGACAAATACGATTTATCTAATTTACACGAAATTGCTTCTGGTGGCGC<br>TCCCCTCTCTAAGGAAGTCGGGGAAGCGGTTGCCAAGAGGTTCCATCTGCCAGGTATCAGGCA<br>AGGATATGGGCTCACTGAGACTACATCAGCTATTCTGATTACACCCGAGGGGGATGATAAACC<br>GGGCGCGGTCGGTAAAGTTGTTCCATTTTTTGAAGCGAAGGTTGTGGATCTGGATACCGGGA<br>AAACGCTGGGCGTTAATCAAAGAGGCGAACTGTGTGTGAGAGGTCCTATGATTATGTCCGGTT<br>ATGTAAACAATCCGGAAGCGACCAACGCCTTGATTGACAAGGATGGATGGCTACATTCTGGA<br>GACATAGCTTACTGGGACGAAGACGAACACTTCTTCATCGTTGACCGCCTGAAGTCTCTGATTA<br>AGTACAAAGGCTATCAGGTGGCTCCCGCTGAATTGGAATCCATCTTGCTCCAACACCCCAACAT<br>CTTCGACGCAGGTGTCGCAGGTCTTCCCGACGATGACGCCGGTGAACTTCCCGCCGCCGTTGT<br>TGTTTTGGAGCACGGAAAGACGATGACGGAAAAAGAGATCGTGGATTACGTCGCCAGTCAAG<br>TAACAACCGCGAAAAAGTTGCGCGGAGGAGTTGTGTTTGTGGACGAAGTACCGAAAGGTCTT<br>ACCGGAAAACTCGACGCAAGAAAAATCAGAGAGATCCTCATAAAGGCCAAGAAGGGCGGAA<br>AGATCGCCGTGTAATTCTAGAGAATTCGATATCATCGCTCGAGGCCGGTCTCCCTATAGTGAG<br>TCGTATTA
firefly luciferase IVT mRNA_HiT7	CAGACATATGCA**TAATACGACTCACTATA**GGGAACCAGATCACTGTGTTTACTTGC<br>AATCCCCCAAAACAGACAGAATGGAAGACGCCAAAAACATAAAGAAAGGCCCGGCGCCATT<br>CTATCCGCTGGAAGATGGAACCGCTGGAGAGCAACTGCATAAGGCTATGAAGAGATACGCCC<br>TGGTTCCTGGAACAATTGCTTTTACAGATGCACATATCGAGGTGGACATCACTTACGCTGAGTA<br>CTTCGAAATGTCCGTTCGGTTGGCAGAAGCTATGAAACGATATGGGCTGAATACAAATCACAG<br>AATCGTCGTATGCAGTGAAAACTCTCTTCAATTCTTTATGCCGGTGTTGGGCGCGTTATTTATC<br>GGAGTTGCAGTTGCGCCCGCGAACGACATTTATAATGAACGTGAATTGCTCAACAGTATGGGC<br>ATTTCGCAGCCTACCGTGGTGTTCGTTTCCAAAAAGGGGTTGCAAAAAATTTTGAACGTGCAA<br>AAAAAGCTCCCAATCATCCAAAAAATTATTATCATGGATTCTAAAACGGATTACCAGGGATTTC<br>AGTCGATGTACACGTTCGTCACATCTCATCTACCTCCCGGTTTTAATGAATACGATTTTGTGCCA<br>GAGTCCTTCGATAGGGACAAGACAATTGCACTGATCATGAACTCCTCTGGATCTACTGGTCTG<br>CCTAAAGGTGTCGCTCTGCCTCATAGAACTGCCTGCGTGAGATTCTCGCATGCCAGAGATCCTA<br>TTTTTGGCAATCAAATCATTCCGGATACTGCGATTTTAAGTGTTGTTCCATTCCATCACGGTTTT<br>GGAATGTTTACTACACTCGGATATTTGATATGTGGATTTCGAGTCGTCTTAATGTATAGATTTG<br>AAGAAGAGCTGTTTCTGAGGAGCCTTCAGGATTACAAGATTCAAAGTGCGCTGCTGGTGCCAA<br>CCCTATTCTCCTTCTTCGCCAAAAGCACTCTGATTGACAAATACGATTTATCTAATTTACACGAA<br>ATTGCTTCTGGTGGCGCTCCCCTCTCTAAGGAAGTCGGGGAAGCGGTTGCCAAGAGGTTCCAT<br>CTGCCAGGTATCAGGCAAGGATATGGGCTCACTGAGACTACATCAGCTATTCTGATTACACCC<br>GAGGGGGATGATAAACCGGGCGCGGTCGGTAAAGTTGTTCCATTTTTTGAAGCGAAGGTTGT<br>GGATCTGGATACCGGGAAAACGCTGGGCGTTAATCAAAGAGGCGAACTGTGTGTGAGAGGT<br>CCTATGATTATGTCCGGTTATGTAAACAATCCGGAAGCGACCAACGCCTTGATTGACAAGGAT<br>GGATGGCTACATTCTGGAGACATAGCTTACTGGGACGAAGACGAACACTTCTTCATCGTTGAC<br>CGCCTGAAGTCTCTGATTAAGTACAAAGGCTATCAGGTGGCTCCCGCTGAATTGGAATCCATC<br>TTGCTCCAACACCCCAACATCTTCGACGCAGGTGTCGCAGGTCTTCCCGACGATGACGCCGGT<br>GAACTTCCCGCCGCCGTTGTTGTTTTGGAGCACGGAAAGACGATGACGGAAAAAGAGATCGT<br>GGATTACGTCGCCAGTCAAGTAACAACCGCGAAAAAGTTGCGCGGAGGAGTTGTGTTTGTGG<br>ACGAAGTACCGAAAGGTCTTACCGGAAAACTCGACGCAAGAAAAATCAGAGAGATCCTCATA<br>AAGGCCAAGAAGGGCGGAAAGATCGCCGTGTAATTCTAGAGAATTCGATATCATCGCTCGAG<br>GCCGGTCTCCCTATAGTGTCACCTAAATCG

Supplementary table 2. Sequences of the templates used for IVT reactions
